# Cell Death Inducing Microbial Protein Phosphatase Inhibitors—Mechanisms of Action

**DOI:** 10.3390/md13106505

**Published:** 2015-10-22

**Authors:** Rune Kleppe, Lars Herfindal, Stein Ove Døskeland

**Affiliations:** 1 Department of Biomedicine, University of Bergen, Jonas Lies vei 81, N-5009 Bergen, Norway; E-Mails: lars.herfindal@uib.no (L.H.); stein.doskeland@uib.no (S.O.D.); 2 Department of Clinical Science, University of Bergen, Jonas Lies vei 65, N-5021 Bergen, Norway

**Keywords:** microcystin, okadaic acid, nodularin, cell death, apoptosis, protein phosphatase, inhibitor

## Abstract

Okadaic acid (OA) and microcystin (MC) as well as several other microbial toxins like nodularin and calyculinA are known as tumor promoters as well as inducers of apoptotic cell death. Their intracellular targets are the major serine/threonine protein phosphatases. This review summarizes mechanisms believed to be responsible for the death induction and tumor promotion with focus on the interdependent production of reactive oxygen species (ROS) and activation of Ca^2+^/calmodulin kinase II (CaM-KII). New data are presented using inhibitors of specific ROS producing enzymes to curb nodularin/MC-induced liver cell (hepatocyte) death. They indicate that enzymes of the arachidonic acid pathway, notably phospholipase A2, 5-lipoxygenase, and cyclooxygenases, may be required for nodularin/MC-induced (and presumably OA-induced) cell death, suggesting new ways to overcome at least some aspects of OA and MC toxicity.

## 1. Introduction

The dinoflagellate toxin okadaic acid (OA) and the toxic cyanobacterial microcystins (MC, including nodularin), pose a health hazard to both humans and livestock [1]. Although OA and MC/Nod are produced by organisms from two different kingdoms and have disparate primary structure, their main target is the same; phospho-Ser/-Thr protein phosphatases (PPs), foremost the PP1s and PP2As of this PP class. The targeted PPs are pivotal in eukaryote signaling, where they are controlled by hundreds of distinct regulatory scaffolding subunits as well as endogenous inhibitory peptides [2,3].

## 2. Structural Features of Microbial Toxins

**Figure 1:**
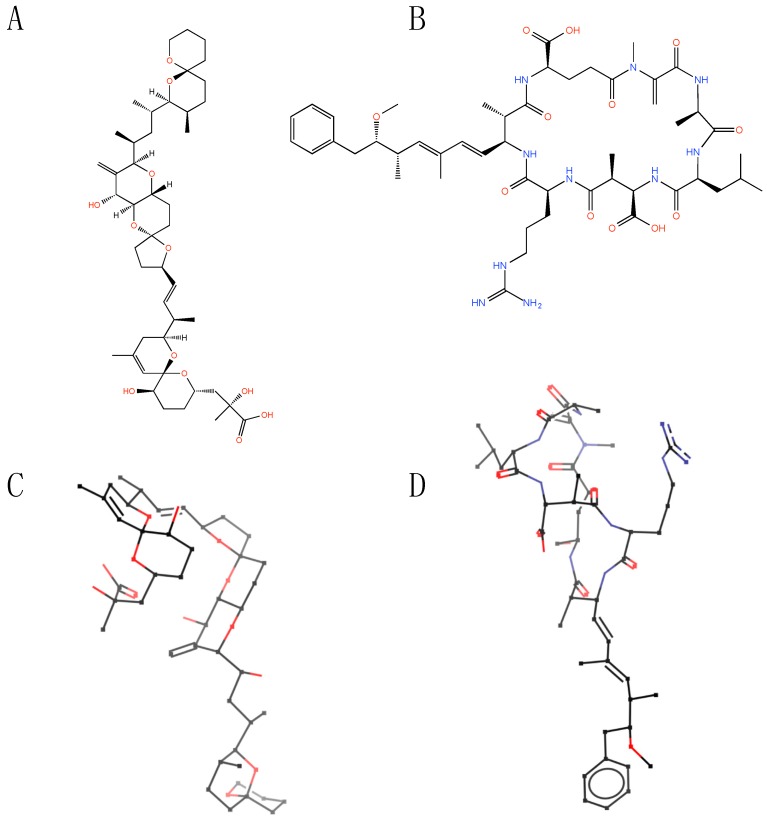
(**A**,**B**) Structure of okadaic acid (**A**) and microcystin (**B**); (**C**,**D**) the three-dimensional representation of okadaic acid (**C**) and microcystin (**D**) when complexed with protein phosphatase 1 (PP1). The OA-PP1structure is from [14], and the MC-LR-PP1 structure is from [13].

OA and MC are relatively complex structures (Figure 1A,B), with a molecular weight of about 800 and 1000 Da, respectively. OA is a polyether with a terminal carboxy-moiety [4]. MC is a family of cyclic heptapeptides [5,6] with a general structure of cyclo-(d-Ala1-Xaa2-d-MeAsp3-Yaa4-Adda5-d-Glu6-Mdha7) where the amino acids in the two and four position are variable. The most common structure is MC-LeuArg (MC-LR), but other variants such as TyrArg, LeuTrp, and LeuAla are also common. MC contains several non-proteinogenic amino acids, such as Adda (3-amino-9-methoxy-2,6,8-trimethyl-10-phenyl-4,6-decadienoic acid), d-MeAsp (d-erythro-β-methylaspartic acid), and Mdha (*N*-methyldehydroalanine). The related PP inhibiting cyanobacterial cyclic pentapeptide nodularin (Nod) shares several non-proteinogenic amino acids with MC, such as Adda and d-MeAsp, but instead of Mdha, the dehydro-amino acid Mdhb (2-(methylamino)-2-dehydrobutyric acid) [7,8,9].

The striking similar action of these distinct compounds, which differ in primary structure, becomes understandable upon inspection of their in solution structure, and their docking into the PP active site (Figure 1A,B). The structures of the two toxins in complex with PP1 reveal similar three-dimensional conformation (Figure 1C,D). Either compound has important interactions with the hydrophobic groove and the β12β13-loop of PP, and covers the catalytic site (for details of the toxin binding to PP1 or PP2A see: [10,11,12,13,14]). An interesting feature of MC is the ability of its methyl-dehydroalanine moiety to form a covalent linkage with Cys273 on PP1, leading to irreversible phosphatase inhibition [15]. Nodularin has a similar inhibitory profile as microcystin [16], but the replacement of the *N*-methyldehydroalanine by *N*-methyldehydrobutyrine prevents the formation of a covalent linkage with Cys273.

## 3. Comparative Activity of OA and MC-LR against Various Protein Phosphatases 

OA and MC-LR have been compared for their ability to inhibit a number of PPs (see Table 1 for reported IC_50_ values). MC-LR has high potency (IC_50_ ≤ 1 nM) against PP1, PP2A, PP3, PP4 and PP5, and may therefore be the inhibitor of choice to obtain a more generalized PP inhibition (note, however, that neither OA nor MC show significant inhibition of PP2B/calcineurin, PP7, or PP2C (Table 1). OA shows about 100-fold preference for PP2A and PP4 compared to PP1 (Table 1). Effects obtained at low OA concentrations are therefore unlikely to be caused by PP1 inhibition.

**Table 1:** Reported IC_50_ values (nM) of okadaic acid and microcystin LR for inhibition of various serine-threonine PP family members.

PP family Member ^1^	IC_50_ (nM)			
	Okadaic acid	References	MC-LR	References
PP1	15–50	[17,18]	0.3–2	[15,17,18,19,20]
PP2A	0.1–0.3	[17,18]	0.05–1	[17,18,19,20]
PP2B	~4000	[17]	~2000	[17,19]
PP3	3.7–4	[18]	0.2	[18]
PP4	0.1	[17,21]	0.15	[17,21]
PP5	3.5	[17]	1.0	[17]
PP7	>1000	[17,22]	>1000	[17,22]

^1^ Data for PP2C (a member of the PPM family of phosho-protein phosphatases) have, to the best of our knowledge, not been published, but one report states that nodularin lacks activity towards this phosphatase [16].

Most inhibition assays require a substantial concentration of phosphatase (typically near 1 nM) to achieve significant dephosphorylation. This may lead to under-estimation of the IC_50_ of unbound toxin (see [23] for details). Data obtained by a competition assay between various toxins and [^125^I]-labeled MC-YR at very low PP2A concentrations (Table 2) illustrate this point. This assay reveals that MC-LR binds with even higher affinity (IC_50_ = 0.017 nM) to PP2A (Table 2) than revealed by inhibition assays (Table 1). Note also that nodularin is a high affinity PP2A inhibitor (IC_50_ = 0.015 nM) and that tautomycin, often considered a preferential PP1 inhibitor [19], has IC_50_ < 0.6 nM for PP2A (Table 2).

**Table 2:** IC_50_ values for various phosphatase inhibitors for binding to PP2A.

Compound	IC_50_ (nM)
Nodularin	0.015
MC-LR	0.017
MC-YR	0.075
OA	0.100
CalyculinA	0.250
Tautomycin	0.560

Data are from [23], and are based on competitive displacement of [^125^I]-labeled MC-YR from purified PP2A.

## 4. Cellular Uptake of Microbial Phosphatase Inhibitors

The mechanisms of how OA and MC toxins enter cells are fundamentally different. Okadaic acid is amphiphilic [24] enough to cross membranes. Membrane interactions of MC have been reported [25], likely through the insertion of the hydrophobic Adda moiety. MC lacks, however, the ability of the lipopeptide anabaenolysin to permeabilize membranes in a cholesterol-dependent manner [26]. In fact, MC requires transporter proteins to enter cells [27,28]. This may be due to both its charge and polarity and its bulk, since addition of a hydrophobic acyloxy-methyl ester on the carboxyl-groups or phenyl-glyoxal on the arginine of MC or nodularin did not improve significantly the entry into cells lacking MC transporters [29]. As a consequence of their different membrane permeability OA can induce cell death with apoptotic features [30] in virtually all cell types, whereas MC only induces apoptosis in cells expressing the liver specific organic anion transporter polypetides (OATP) 1B1 or 1B3 [28]. For cell experiments, this means that only primary hepatocytes, or cells with enforced expression of the transporters are sensitive to MC at nM concentrations, while mammalian cells not expressing OATP1B1/3 require concentrations above 10 μM [31,32,33]. For this reason isolated primary hepatocytes have been used as an alternative to mouse survival test to detect MC/nodularin or diarrhetic shellfish poisons like OA, using the rapid induction of apoptosis as read-out [34].

MC and nodularin depend on hepatocyte OATP1B1/3 for cell entry [27] OATP1B1/3 inhibitors will protect hepatocytes against both nodularin and MC induced apoptosis [35]. It is of interest that some cyanobacterial nostocyclopeptides are OATP1B1/3 inhibitors whose original function may have been to block nutrient channels of their primitive eukaryotic predators [36]. One may consider MC and nodularin as competitive channel blocking toxins that have acquired the additional property of inhibiting the major PPs in predator cells. The extreme conservation of the active site of PP2A and PP1 through evolution explains why these ancient toxins also act on human phosphatases.

## 5. Features of Microbial PP Inhibitor-Induced Cell Death

As first reported in [30] the phosphatase inhibitors OA and MC-LR induce cellular apoptosis. The apoptosis was independent of new protein synthesis and therefore not mediated by the execution of a genetic program. The OA-or MC/nodularin-induced death had classical morphological features of apoptosis such as cell and nuclear shrinkage, chromatin condensation, formation and shedding of apoptotic bodies, annexin-V externalization, but lacked DNA fragmentation [30,37,38]. Internucleosomal DNA fragmentation and 28S ribosomal RNA cleavage was observed in some, but not all, OA-treated leukemia cell lines, suggesting that this trait is cell type specific [39,40].

Secondary necrosis (apo-necrosis) is a late phenomenon in MC or nodularin exposed hepatocytes [38]. In contrast, we have observed that primary hepatocytes exposed to hypoxia during isolation responded to MC/nodularin with primary necrotic death, characterized by membrane leakage and secondary DNA fragmentation. This is reminiscent of the switch from an apoptotic to a necrotic response to tumor necrosis factor α in cells with depleted energy stores or inactive caspase 8 (see [41] for recent review). Inactive caspase 8 cannot alone explain the necrotic phenotype of hypoxic hepatocytes since the caspase inhibitor z-VAD-fmk had little effect on MC-induced hepatocyte apoptosis in normoxic hepatocytes [37]. The features of PP-inhibitor-induced death depend not only on the target cell and its energy state, but also on the concentration and nature of the inhibitor. Thus, while acute myeloid leukemia (AML) cells incubated with high concentration of calyculin A (CalA) lacked the nuclear envelope and had increased ATP content, OA or CalA at low concentration induced DNA and RNA cleavage, nuclear fragmentation, and cell shrinkage [42].

## 6. The Pathogenesis of the Acute Hepatotoxicity of MC and Nodularin

The ingestion of MC or Nod producing cyanobacteria can cause lethal acute liver damage. The death occurs too rapidly to be explained by hepatocyte metabolic failure alone, and is probably due to disintegration of the liver micro-architecture, accompanied by lethal bleeding [43,44]. As first shown by Eriksson [45], MC causes strong condensation of hepatocyte actomyosin filaments accompanied by blebbing [46]. Cell blebbing was also noted in an early study by Runnegar *et al*. [47]. The actomyosin contraction may be related to myosin light chain hyperphosphorylation, which coincides with cell shrinkage and blebbing, and is one of the first phosphorylation events detected in hepatocytes exposed to MC [37]. Both shrinkage and blebbing depend on high intracellular pressure, which can be produced by general hypercontraction of the subplasmalemnal actomyosin web.

Loosening of cell-cell and cell-substratum contacts has been observed in hepatocyte monolayers [48]. In fact, hepatocytes exposed for only 30 min to 0.5 µM MC-LR remained irreversibly shrunk and blebbed, and were unable to attach to collagen substratum [38]. The combination of hepatocyte shrinkage and detachment is expected to disrupt bile canaliculi and blood vessels, resulting in lethal leakage of blood and toxic bile acids. The PP-inhibitor responses noted above are not unique for hepatocytes [49].

It is questionable if mitochondrial are early targets in PP-inhibitor induced cell death. Thus, OA or CalA failed to synergize with cAMP to induce cell death in AML cells [42], in which cAMP acts by inducing the expression of the pro-apoptotic Bcl-2 protein Bim, with subsequent mitochondrial damage [50]. Additionally, cells overexpressing the mitochondria-protecting B-cell lymphoma (Bcl)-2 or Bcl-XL were equally sensitive to microinjected nodularin as wt cells [37]. Micro-mitochondriosis, induced by mitochondrial fission, has, however, been observed in AML cells exposed to high concentration of CalA [42] and in neurons exposed to OA [51].

## 7. PP Inhibitors as Tumor Promoters

OA became early known as a promoter of skin tumors [52], and MC as a potential hepatocarcinoma promoter [53]. The discovery of the mitogen activated protein kinase (MAPK) cascade and its antagonism by PP2A, and that the oncogenic SV40 small T antigen was a PP2A inhibitor [54,55], led to the assumption that OA and MC tumor promotion was mediated mainly by stimulation of MAPK to enhance cell proliferation and survival. The assumption is supported by studies showing that CalA-enhanced ERK activity may protect U937 cells against death [56] and that MC may enhance slightly hepatocyte proliferation [57]. OA can, however, even at sub-apoptotic concentration, inhibit cellular DNA replication [57,58]. The dualistic effects of MC-LR and OA have been reviewed by Gehringer [59]. It is also possible that PP inhibitor induced cell damage leads to compensatory growth of less damaged neighboring cells. Other mechanisms whereby OA can promote tumor formation are by interference with normal centrosome duplication and chromosome segregation during mitosis [60,61].

## 8. The Role of CaM-KII in PP Inhibitor Action—Is CaM-KII an Amplifier of ROS Generation?

Phosphatase inhibition can increase phosphorylation of a protein only if the relevant protein kinase(s) is active. The ubiquitously expressed Ca^2+^/calmodulin dependent protein kinase II (CaM-KII) was found essential for OA-, MC- and nodularin- induced apoptosis and early phosphorylation events in a number of cell types, as well as for the OA-induced inhibition of hepatocyte DNA replication [38,57,62]. Since CaM-KII autophosphorylation on Thr286 [63] is reversed by several phosphatases, including PP1 and PP2A [63], it is expected that PP inhibitors activate CaM-KII by enhancing the activating autophosphorylation on Thr286 [63]. More surprising is that CaM-KII inhibitors also can block PP inhibitor induced protein phosphorylation events catalyzed by other protein kinases. One mechanism of CaM-KII action is through direct activating phosphorylation of other kinases such as myosin light chain kinase [38,62], which may explain the early hyperphosphorylation of myosin light chain in MC- or nodularin- exposed cells [38,62]. Another mechanism is through generation of reactive oxygen species (ROS), which also feedback on CaM-KII itself as it is subject to activation by oxidation of Met281/282 [64].

Protein phosphatases are inhibited by ROS that target specific redox sensitive amino acids. The reversible inhibitory oxidation of protein tyrosine phosphatases by hydrogen peroxide was discovered in the mid-1990s, whereas redox regulation of Ser/Thr phosphatases is a more recent discovery [65]. The PP2A regulatory subunit B56δ is reported to become tyrosine nitrated, leading to poor holoenzyme formation and substrate targeting [66]. The direct inhibition of PP2A catalytic activity by hydrogen peroxide has also been reported, leading to the activation of cellular stress signaling pathways [67].

**Figure 2:**
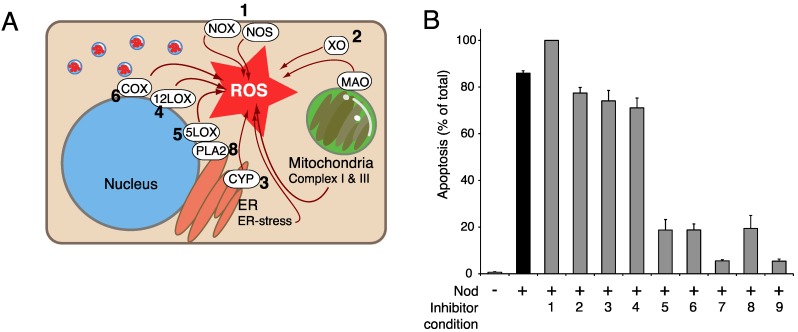
ROS-producing enzymes in PPI-induced apoptosis. (**A**) Illustrates some of the major cellular sources of reactive oxygen species (ROS). The illustration focuses on different ROS producing enzymes; NADPH oxidase (NOX), nitric oxide synthase (NOS), xanthine oxidase (XO), monoamine oxidase (MAO), cyclooxygenases (COX), 12-Lipoxygenase (12LOX), 5-Lipoxygenase (5LOX), and microsomal Cytochrome P450 complexes (CYP). Cellular phospholipase A2 (PLA2) does not generate ROS, but acts upstream of COX and 5LOX. The numbers in bold refers to the enzyme that is targeted by the inhibitors used in panel. (**B**). The effect of different inhibitors towards ROS producing enzymes was tested against nodularin (Nod, 100 nM, 75 min) induced apoptosis in isolated primary rat hepatocytes. The inhibitors (assigned with numbers) were: (1) Diphenyleneiodonium chloride (25 µM), (2) allopurinol (100 µM), (3) metyrapone (500 µM), (4) baicalein (5 µM), (5) AA861 (5 µM), (6) Indomethacine (10 µM), (7) nordihydroguaiaretic acid (20 µM), (8) arachidonyl trifluoromethylketone (5 µM), and (9) naringin (100 µM). After treatment, the cells were fixed evaluated for apoptosis and necrosis by microscopy. Treatment with nodularin gave rise to 86% apoptosis, compared to less than 1% in control.

Several cellular activities can produce ROS (Figure 2A) and are, therefore, potential sources of the ROS increase by PP inhibitors. The extent to which locally produced ROS accumulates will depend on the presence of nearby enzymes such as superoxide dismutase, catalase, the selenocysteine containing glutathione peroxidases and thioredoxin reductases, which catalyze the conversion of different ROS types to harmless molecules. In addition, glutathione, *N*-acetyl-cysteine and redox active proteins like peroxyredoxins and methionine sulfoxide reductases can facilitate the normalization of ROS modified proteins. PP inhibitors can therefore enhance ROS accumulation also by inhibiting the action of ROS scavengers or neutralizers.

The oxidative metabolism in mitochondria forms superoxide [68] that can be transported through voltage dependent anion channels to the cytosol [69] unless converted to freely diffusible H_2_O_2_ by mitochondrial superoxide dismutase. Mitochondria may not be the major primary target of CaM-KII mediated PP-inhibitor induced death as little impairment of nodularin-induced apoptosis was observed in hepatocytes pretreated (5 min) with inhibitors against mitochondrial complex I and III (rotenone and azide). Increased mitochondrial ROS has been reported in neurons undergoing OA-induced death [51]. Mitochondria can also be affected downstream of the ROS/CaM-KII-dependent activation of the apoptosis signaling kinase 1, which leads to activation of the c-Jun *N*-terminal Kinase and p38-MAPKinase apoptotic pathways [70,71,72,73].

The plasma membrane NADPH-oxidase generates superoxide, normally through stimulation by Rac [74], but also through stimulation by okadaic acid [75]. In our hands NADPH-oxidase inhibitor failed to prevent nodularin-induced hepatocyte apoptosis (Figure 2B).

The activity of oxygenases like xanthine oxidase (XO) and monoamine oxidases (MAO) can produce both peroxide and superoxide [76,77]. The aromatic amino acid hydroxylases form H_2_O_2_ when the oxidation of substrate is uncoupled [78,79], as when the enzyme binds the tetrahydrobiopterin cofactor without amino acid substrate [80]. It is of interest that CaM-KII phosphorylation of phenylalanine hydroxylase is enhanced in OA-exposed hepatocytes when the hydroxylase does not bind phenylalanine [81], suggesting that CaM-KII selectively activates the hydroxylase in a state prone to produce ROS rather than to hydroxylate phenylalanine.

Cytochrome P450 monooxygenases (CYP) can also generate ROS during conditions of poor coupling. The NADPH-CYP reductase and cytochrome b5 is a major source of ER-generated ROS [82]. Other known sources of ROS are nitric oxide synthase (NOS) [83] and enzymes of lipid metabolism such as lipoxygenases (LOXs) and cyclo-oxygenases (COXs) [84,85]. We have previously reported the involvement of CaM-KII and ROS in PPI-induced hepatocyte apoptosis [38,86]. To trace the source of the ROS we tested inhibitors of various ROS-producing enzymes, listed above, for ability to inhibit the nodularin-induced apoptosis of freshly isolated rat hepatocytes. The xanthine oxidase inhibitor allopurinol and the CYP inhibitor metyrapone interfered only slightly with the nodularin-induced apoptosis, while the NO synthase (NOS)/NADPH oxidase inhibitor diphenyleneiodonium, if anything, enhanced slightly the apoptosis (Figure 2B). We considered therefore if the nodularin could act by enhancing ROS production through the phospholipase A2/LOX/COX pathway. This pathway has been implicated in neuronal cell death [87], neurodegeneration and glutamate toxicity [88,89] and its superoxide production may be auto-amplified by ROS-induced phospholipase A2 (PLA2) activation [90].

## 9. The Potential Role of Perturbed Phospholipid Metabolism in PPI-Induced Hepatocyte Apoptosis

Inhibitors of cyclooxygenases COX1 and 2 (indomethacine) and against LOXs in general (nordihydroguaiaretic acid) were potent antagonists of nodularin-induced apoptosis. Interestingly, the 5-lipoxygenase (5-LOX) inhibitor AA861 protected against nodularin while the 12-lipoxygenase (12-LOX) inhibitor baicalein did not (Figure 2B). Although some of the inhibitors may have off-targets effects, like general anti-oxidant effects for nordihydroguaiaretic acid, the strong effect of all tested inhibitors of the PLA2/5-LOX/COX pathway is striking. These findings supplement the few previous reports connecting this pathway with PP inhibitors or CaM-KII [91,92].

It should be noted that okadaic acid enhances cellular CaM-KII catalyzed phosphorylation and activation of PLA2, which subsequently translocates from cytoplasm to perinuclear membranes [93], where it can co-localize with COX1 in the trans-Golgi network [94]. 5-LOX is presumably most active when complexed with the nuclear envelope and endoplasmic reticulum located 5-LOX activating protein (FLAP) and the leukotriene-C4 synthase [95] (see also [96] for a review). When phosphorylated by CaM-KII the 5-LOX can shift from a cytoplasmic to a nuclear location [97]. This may be significant because OA-induced cell death is counteracted by the perinuclearly located GTPase of the immune-associated proteins 5 (Gimap5) protein [98].

## 10. Gimap5 (Ian5, IROD) is an Inhibitor of OA-Induced Apoptosis

Gimap5, also known as Ian5 and IROD, is located in the perinuclear centrosomal/Golgi/ER compartment and its coiled-coil domain is essential for protection against both OA and acute ionizing radiation [98]. It was found by screening fibroblasts transduced with a protein expression library for survival at high OA concentration, and does not protect against microinjected cytochrome C, Fas-ligation, UV radiation, serum deprivation, or a number of chemotherapeutic drugs [98,99]. The major pathology in Gimap5 knock-out mice is decreased survival of T cells and natural killing cells as well as hepatocyte apoptosis and liver failure leading to death after about 15 weeks of life [100]. These findings imply that the perinuclear region where Gimap5 resides may be particularly vulnerable to the acute apoptotic death induced by PP inhibitors, also in hepatocytes, thus reinforcing the significance of the perinuclearly located ROS generating enzymes found required for PP inhibitor induced hepatocyte death (Figure 2B).

## 11. Concluding Remarks

In addition to their obvious health consequences, the marine protein phosphatase targeting toxins like okadaic acid give insight into cell death mechanisms. It is particularly intriguing that major actions of the protein phosphatase inhibiting toxins are mediated by CaM-KII in conjunction with increase of cellular ROS, possibly primarily in the perinuclear region of the target cell.

## Abbreviations

5-LOX—5-lipoxygenase; 12-LOX—12-lipoxygenase; Adda—3-amino-9-methoxy-2,6,8-trimethyl-10-phenyl-4,6-decadienoic acid; AML—acute myeloid leukemia; Bcl—B-cell lymphoma; CaM-KII—Ca^2+^/calmodulin dependent protein kinase II; CalA—calyculin A; COX—cyclo-oxygenase; CYP—Cytochrome P450 complex; d-MeAsp—D-erythro-β-methylaspartic acid; Gimap5—GTPase of the immune associated protein family 5; MAO—monoamine oxidase; MAPK—mitogen activated protein kinase; MC—microcystin; Mdha—*N*-methyldehydroalanine; Mdhb—2-(methylamino)-2-dehydrobutyric acid; Nod—nodularin; NOX—NADPH oxidase; NOS—nitric oxide synthase; OA—okadaic acid; OATP—organic anion transporter polypeptide; PLA2—phospholipase A2; PP—protein phosphatase (of the phospho-Ser/-Thr phosphatase family); ROS—reactive oxygen species; XO—xanthine oxidase.
